# Retro-Mode in NIDEK Mirante: A Comparative Analysis with Other Imaging Modalities for AMD and CSR

**DOI:** 10.3390/diagnostics13172846

**Published:** 2023-09-02

**Authors:** Adam Wylęgała, Przemysław Wozniak, Bogumiła Sędziak-Marcinek, Bartłomiej Bolek, Dominika Szkodny, Edward Wylęgała

**Affiliations:** 1 Health Promotion and Obesity Management, Pathophysiology Department, Medical University of Silesia in Katowice, 40-752 Katowice, Poland; 2 Chair and Clinical Department of Ophthalmology, School of Medicine in Zabrze, Medical University of Silesia in Katowice, District Railway Hospital, 40-760 Katowice, Poland

**Keywords:** retro-mode, Nidek, FAF, multimodal imaging, Mirante

## Abstract

Background: Retro-mode is a novel technique capable of creating pseudo-3D images of the retina. However, its clinical utility remains unknown. This study aimed to evaluate the Nidek Mirante multimodal imaging platform for ocular assessment in patients with various retinal conditions. Methods: A total of 115 participants with central serous chorioretinopathy (CSR) and age-related macular degeneration (AMD) were included. Two experienced graders independently evaluated the images, and statistical analysis was performed to assess interclass correlation coefficients (ICC) between graders and modalities; Results: For CSR detection, retro-mode demonstrated exceptionally high ICC rates (ICC = 1; 100%), while color and autofluorescence (FAF) showed moderate coefficients (0.69 and 0.78, respectively). The detection of pigment epithelial detachment was high across all methods, with only retro-mode deviated right (DR) allowing detection in 69% of cases, while retro-mode DR and deviated left (DL) achieved 100% detection. FAF-green achieved a 95% detection rate. In detecting retinal atrophy, most modalities demonstrated high detection rates, with the lowest detection rates offered by retro-mode DL (ICC = 0.85) and DR (ICC = 0.89), while retro-mode ring aperture offered 0.97. Infra-red and fluorescein angiography imaging offered the highest detection rates among the tested modalities, with 97% and 100%, respectively. Conclusion: Retro-mode showed promise for comprehensive ocular evaluation and diagnosis, with certain imaging modalities demonstrating higher accuracy in detecting specific retinal features.

## 1. Introduction

Imaging plays a crucial role in ophthalmic diagnosis and management, enabling the detection and diagnosis of eye regions not easily accessible through simple examination. These techniques document and measure conditions, making patient follow-ups more reproducible. The utilization of various imaging modalities in daily ophthalmological practice enhances the ability to detect and track pathology, stage diseases, and determine appropriate treatment options and prognoses [1,2].

Commonly employed imaging modalities include optical coherence tomography (OCT), fluorescein angiography (FA), indocyanine green angiography (ICGA), and scanning laser ophthalmoscopy (SLO). SLOs are frequently used to obtain standard reflectance monochromatic or multiwavelength fundus images. Some SLOs offer a retroillumination mode (retro-mode), generating ‘pseudo-three-dimensional’ (pseudo-3D) pictures [3].

As a noninvasive angiographic approach, OCT angiography (OCTA) facilitates the observation of vascular structures. Other enticing technologies, including adaptive optics (AO), ultra-widefield imaging, fundus autofluorescence (FAF), and photoacoustic microscopy (PAM), have been have been integrated into existing retinal imaging modalities, offering the potential to greatly enhance image quality and expand the field of view [1,4,5].

These imaging techniques capture a huge number of detailed images of the retina that allow practitioners to identify small alterations at a high resolution [6]. However, the number of captured images makes manual analysis time consuming and difficult during clinical practice.

Scanning laser ophthalmoscopes (SLOs) typically record images through the direct mode, capturing light retroreflected from chorioretinal structures. However, in the retro-mode (indirect mode), SLOs use light or infrared radiation that transilluminates chorioretinal structures after passing through deeper choroidal and scleral layers [7].

Recently, retro-mode imaging, based on retro-illumination concepts, has been applied to analyze numerous retinal disorders, including deep retinal diseases and retinal pigment epithelium (RPE) alterations. In this method, images are formed by light transilluminating chorioretinal structures (retro-mode), instead of being reflected from their inner surfaces. Retro-mode also improves the detection rate of large-area chorioretinal abnormalities that are not identified with conventional en-face retinal imaging modalities. One characteristic of retro-mode is the sense of elevation or depression in captured structures, which is not typical in reflectance SLO imaging [8,9,10,11]. The recently released scanning laser ophthalmoscope Nidek Mirante (SLO, Nidek Co., Ltd., Gamagori, Japan) provides a revolutionary noninvasive imaging approach called retro-mode scanning laser ophthalmoscopy. The technique uses retro-mode imaging with an infrared laser (790 nm) to study the deeper retinal layers and RPE [12,13]. However, it is still unknown how this new method compares with the more established imaging techniques.

This study aims to evaluate and compare the effectiveness and clinical utility of retro-mode using the NIDEK Mirante imaging system with other imaging modalities offered by this machine for ocular assessment. The findings of the study will contribute to a better understanding of the capabilities and potential advantages of NIDEK Mirante in ocular imaging, ultimately informing clinical practice and future research in the field.

## 2. Materials and Methods

This study employed a retrospective design to evaluate the NIDEK Mirante (Nidek Co., Ltd., Gamagori, Japan) multimodal imaging platform for ocular assessment. The study was conducted at Railway Hospital Katowice Poland, department of Ophthalmology Railway Hospital Katowice, and it received ethical approval from the Ethics Committee of the Silesian Medical University.

A total of 115 participants were recruited from a database of patients who had been imaged between 2021 and 2023. Inclusion criteria encompassed individuals presenting with various retinal conditions, including age-related macular degeneration (AMD), central serous chorioretinopathy (CSR), and pattern dystrophies. Participants with contraindications to ocular imaging such as media opacities and low-quality (<7/10) or incomplete exam protocols were excluded. Basic ocular function tests are displayed in Table 1. 

The NIDEK Mirante multimodal imaging platform was employed for data acquisition. This system integrates spectral-domain optical coherence tomography (SD-OCT) and scanning laser ophthalmoscopy (SLO) capabilities. The SD-OCT component facilitates high-resolution cross-sectional imaging of the retina, while the SLO module facilitates simultaneous fundus imaging and autofluorescence evaluation.

Image Acquisition: Prior to imaging, participants underwent pupil dilation using Tropicamidum (Polfarma, Warszawa, Poland). Subsequently, they were comfortably seated and instructed to maintain fixation on a specific target. Sequential imaging was performed using the NIDEK Mirante system according to the manufacturer’s guidelines. Each patient had a blue–green–red color photo with resolution of 1536 × 1536 (5 times HD repeats), 3D volumetric scan with dimensions of 12.0 × 9.0 mm and resolution of 1024 × 128 (5 times HD repeats), covering the macular region and peripapillary area. Infrared scan autofluorescence blue and green FAF-B FAF-G with resolutions of 1024 × 1024 (30 times HD repeats), fluorescein angiography with resolution of 1024 × 1024 (15 times HD repeats) and retro-mode with resolution of 1536 × 1536 (30 times HD repeats). Some patients also had other OCT scans like radial or cross scans with size of 12 mm (30 times ultrafine repeats). 

Retro-mode uses infrared laser radiation (790 nm) to produce pseudo-3D images of the retina [8].

Retro-mode has three options depending on the aperture deviation. The aperture can be deviated to the left (DL), to the right (DR), or annular (RA)—using a ring aperture. 

Retro-mode (DR or DL aperture) involves restricting the opening of the ring aperture and displacing it laterally from the confocal light path. The purpose of this lateral deviation is to capture only the backscattered light from a specific direction while blocking the directly reflected light and light scattered from other directions. By employing this smaller aperture, Retro-mode generates higher-contrast retro-mode images with a narrower depth of focus in comparison to dark-field images [9,10].

The RA aperture includes a central circular stop that effectively blocks directly reflected light while permitting more widely scattered light to pass through an annular aperture. As a result, this setup produces low-contrast retro-mode images with reduced intensity and distinct illumination patterns [14].

For SLO imaging, the system employed three laser wavelengths: infrared (790 nm), red (670 nm), green (532 nm), and blue (488 nm). Each laser was coupled to a dedicated sensor, enabling simultaneous acquisition of color fundus images. The field of view was set at 50°, capturing a wide-angle view of the ocular structures. 

FAF provides a unique and valuable way to map retinal metabolic changes and structural alterations that cannot be achieved with conventional color imaging. It works by absorbing incident light in the fundus, exciting molecules that then emit specific-wavelength photons. These emitted photons are captured and processed by a sensor to create a metabolic map of the fundus.

Various FAF platforms, including scanning laser ophthalmoscopes and fundus cameras, offer different advantages and limitations that need to be considered before and after imaging to interpret the images accurately. Clinical studies have demonstrated the growing usefulness of FAF in diagnosing and managing various chorioretinal conditions, such as age-related macular degeneration, central serous chorioretinopathy, retinal drug toxicities, and inherited retinal degenerations like retinitis pigmentosa and Stargardt disease [15,16,17]. 

### Data Analysis

The acquired images underwent a process of anonymization and subsequent export.

**Figure 1:**
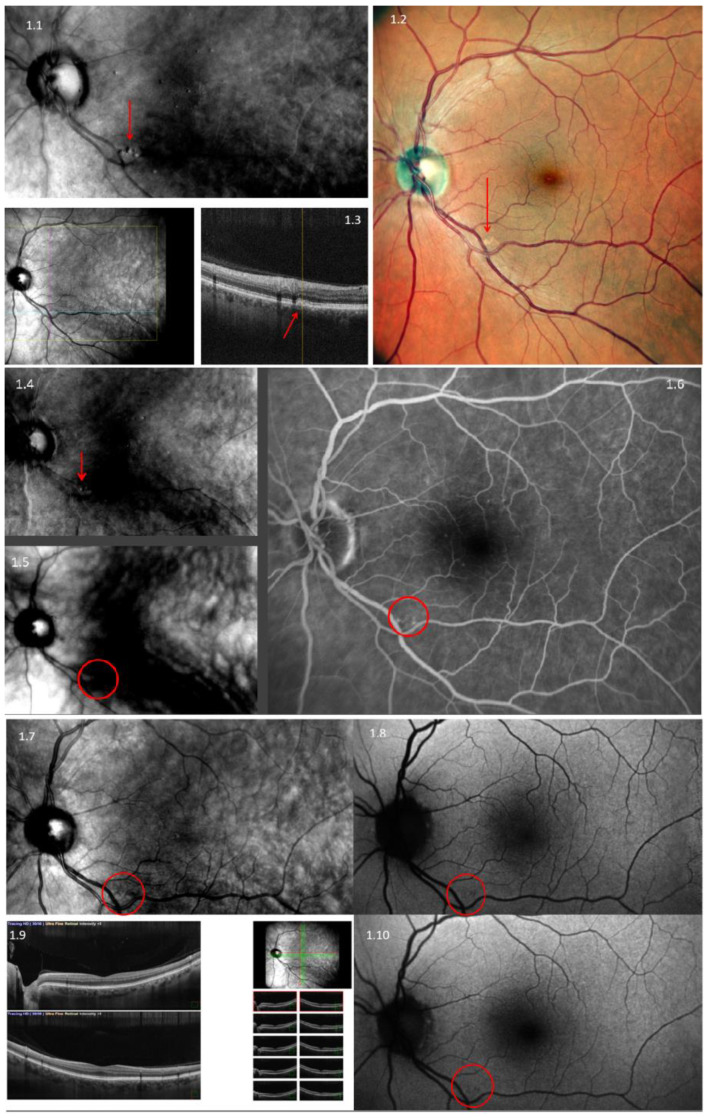
Multimodal imaging of drusen using Nidek Mirante. **1.1**—Retro-mode aperture deviated to the left (DL); **1.2**—color imaging; **1.3**—three-dimensional optical coherence tomography (3D-OCT); **1.4**—retro-mode aperture deviated to the right (DR); **1.5**—retro-mode ring aperture (RA); **1.6**—fluorescein angiography (FA); **1.7**—infrared imaging (IR); **1.8**—fundus autofluorescence–blue-scan (FAF-B); **1.9**-Raster OCT scan; **1.10**—fundus autofluorescence—green-scan (FAF-G). Red arrows and circles denote the presence of drusen.

**Figure 2:**
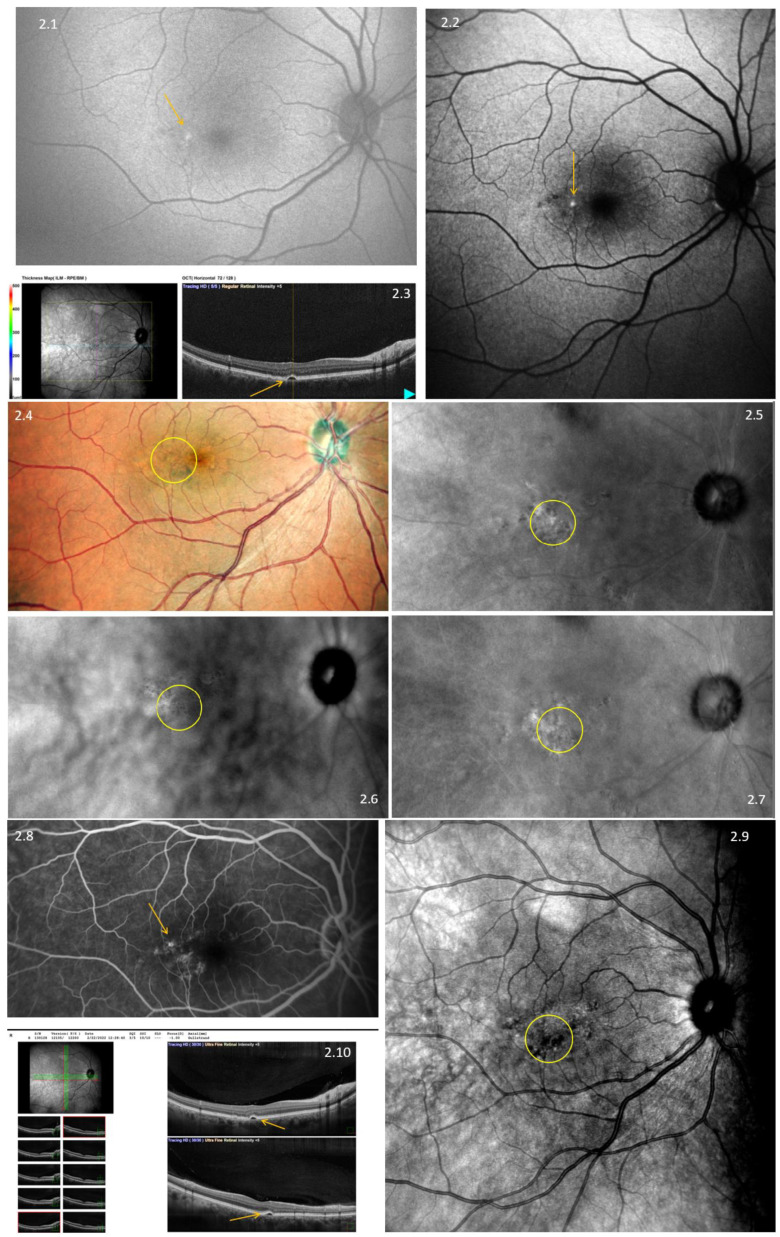
Multimodal imaging of pigment epithelial detachment (PED) using Nidek Mirante. **2.1**—fundus autofluorescence green (FAF-G) grading; **2.2**—fundus autofluorescence–blue-scan (FAF-B); **2.3**—three-dimensional optical coherence tomography (3D-OCT); **2.4**—color imaging; **2.5**—retro-mode aperture deviated to the left (DL); **2.6**—retro-mode aperture deviated to the right (RA); **2.7**—retromode ring aperture (DR); **2.8**—fluorescein angiography (FA); **2.9**—infrared imaging (IR). Yellow arrows and circles denote the presence of PED. **2.10**—raster optical coherence tomography.

**Figure 3:**
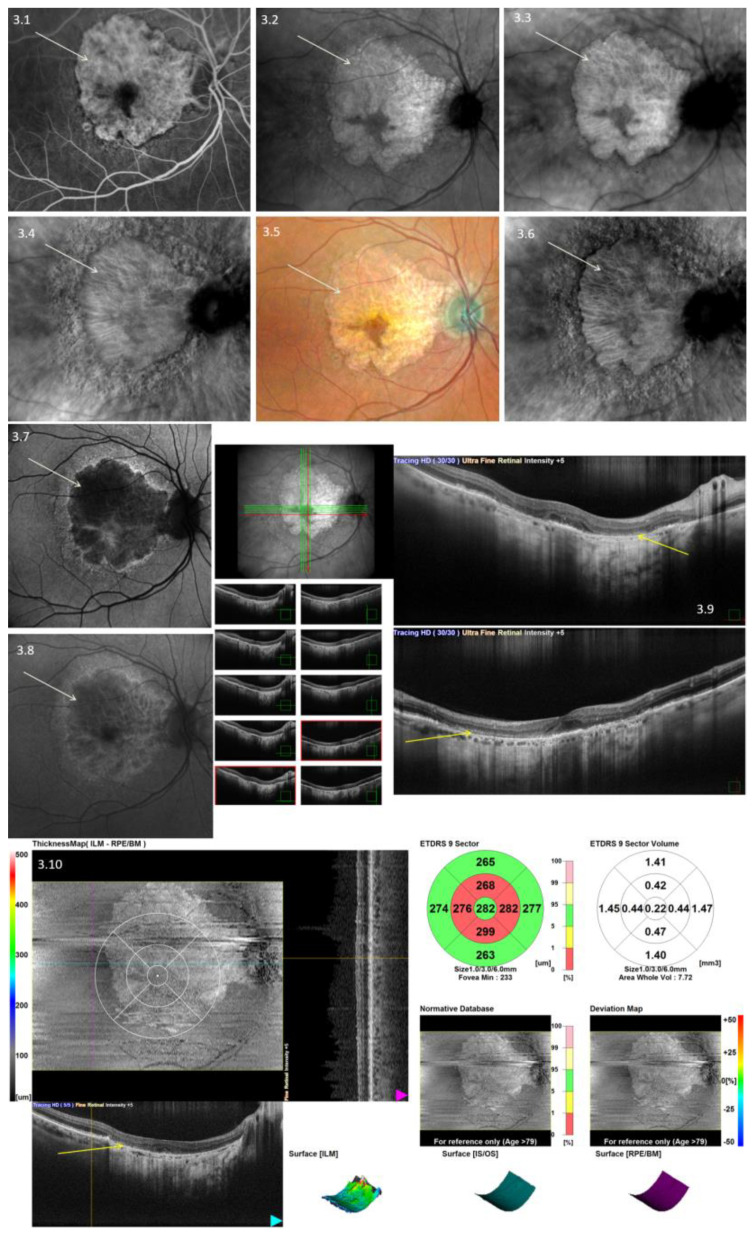
Multimodal imaging of the complete retinal pigment epithelium and ocular retina atrophy (cRORA) using Nidek Mirante. **3.1**—Fluorescein angiography; **3.2**—infrared imaging (IR); **3.3**—retro-mode ring aperture (RA); **3.4**—deviated-left retro-mode aperture (DL); **3.5**—color scanning laser ophthalmoscopy (Color SLO); **3.6**—retro-mode deviated to the right aperture (DR); **3.7**—fundus autofluorescence green (FAF-G); **3.8**—fundus autofluorescence–blue-scan (FAF-B); **3.9**—optical coherence tomography raster-scan (OCT-B scan). **3.10**—optical coherence tomography raster-3-D scan. White arrows show the same spot across multiple scans.

Our data analysis encompassed a comprehensive assessment of image quality, the visualization of anatomical structures, and the identification of four crucial measurement parameters: drusen (as depicted in Figure 1), central serous retinopathy, pigment epithelial detachment (as illustrated in Figure 2), and complete retinal pigment epithelium and outer retinal atrophy (cRORA) (as seen in Figure 3). This analysis was carried out by two experienced graders, who independently evaluated the anonymized images of the retina. Each grading was binary; if any findings were detected, the grader would enter “1” into the spreadsheet; otherwise, they would enter “0”. Their assessments were recorded within an Excel spreadsheet.

Statistical Analysis: We employed descriptive statistics to effectively summarize the demographic characteristics of the study population. Quantitative measurements were rigorously scrutinized using the interclass correlation coefficient (ICC), both between graders A and B and among different imaging modalities. The reference images for this analysis were the OCT scans. The intraclass correlation coefficient (ICC) was calculated for two comparisons: between graders A and B, and between the sum of grades given by graders A and B for a specific modality, as compared to the sum of grades given using OCT (modality vs. OCT).

## 3. Results

This study included 115 participants (68 males and 47 females) with an average age of 61.14 ± 15.61. It comprised 58 right and 57 left eyes, with only one eye selected from each patient. The majority of eyes were diagnosed with dry AMD (42), while macular neovascularization was found in 34 (23 active,11 scar), and CSCR was diagnosed in 31 patients. Additionally, eight individuals had pattern dystrophies. 

**Figure 4:**
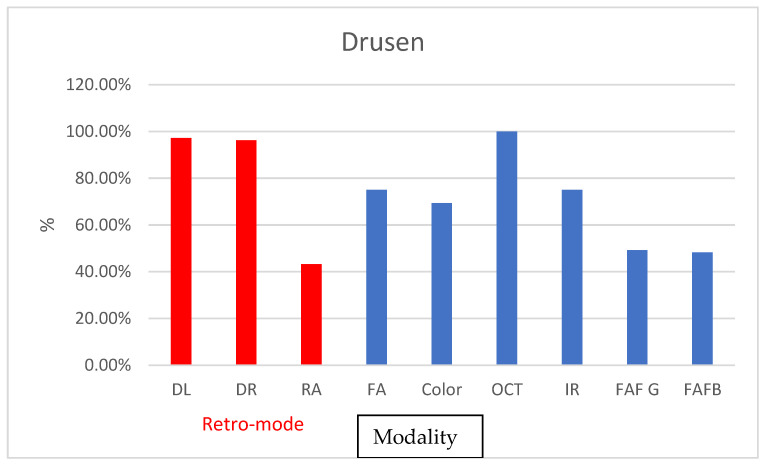
Graph depicting percentage of found cases relative to OCT in pigment epithelial detachment.

For drusen detection, most modalities demonstrated a high level of agreement between graders, with the ICC ranging from 0.83 to 1. However, the ICC values between the modalities and OCT varied from low to high, ranging from 0.39 to 1.00. Overall, both DL and DR exhibited the highest level of agreement among the methods studied. However, for some imaging modalities (RA, Color, FAF-G, and FAF-B), the ICC values were lower, indicating moderate to good agreement between graders in detecting these features. RA was only detected in 43% of drusen cases, while both FAF modalities were successful in almost 50% of cases. In contrast, DR and DL exhibited almost 100% accuracy (Figure 4). 

**Figure 5:**
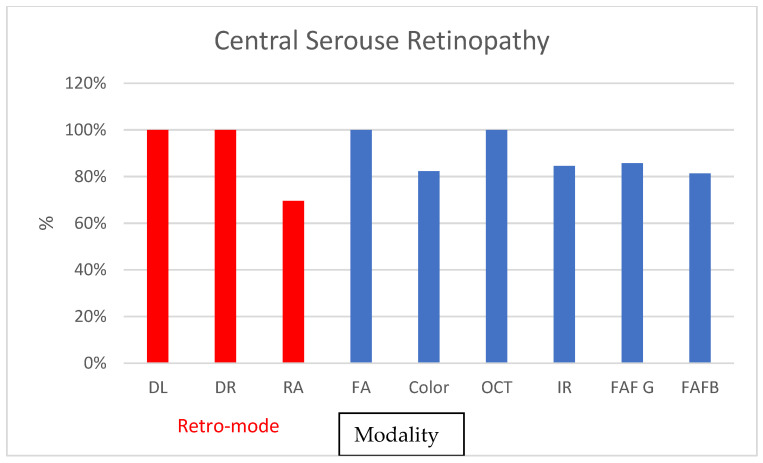
Graphs depicting the percentage of found cases relative to OCT in central serous retinopathy.

DL and DR demonstrated exceptionally high ICC rates for CSR detection (ICC = 1; 100%), while color and FAF showed moderate coefficients (0.69 and 0.78, respectively). The ICC between graders also exhibited high consistency, with RA achieving the lowest ICC of 0.75. RA was effective in only 70% of CSR cases, whereas color SLO achieved detection in 82% of cases. Similar detection rates were observed in FAF-B and IR (Figure 5).

**Figure 6:**
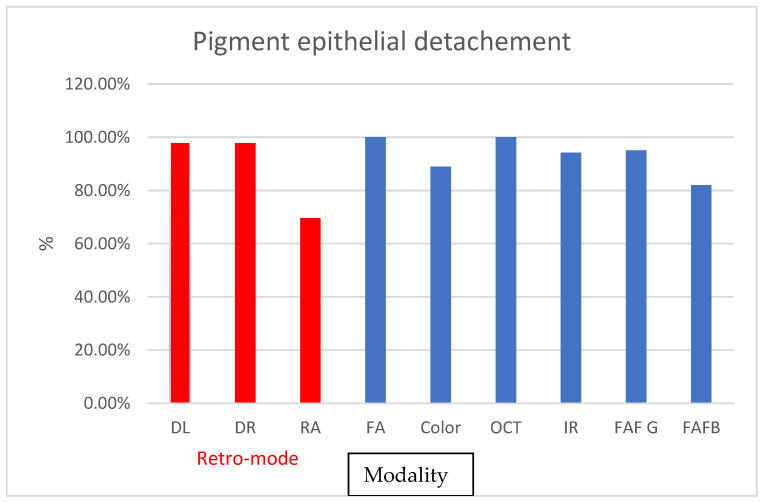
Graphs depicting the percentage of found cases relative to OCT in pigment epithelial detachment.

The detection of PED was high across all utilized methods, with only DR allowing detection in 69% of cases (ICC = 0.5). FA, DL, and DR showed 100% accuracy. FAF-B had an ICC of 0.79 in 82% of cases, while FAF-G achieved 95% accuracy (Figure 6). Interoperator detection rate was also high, with the lowest value for IR being 0.84 and for RA being 0.80.

**Figure 7:**
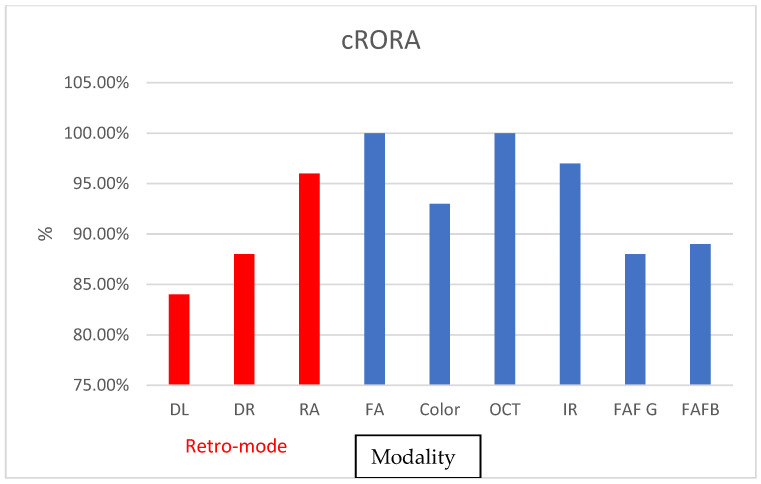
Graphs depicting the percentage of found cases relative to OCT in central serous retinopathy, drusen pigment epithelial detachment, and cRORA Complete RPE and Outer.

cRORA was found to be easily detected with most of the modalities, with the lowest detection rates offered by DL (ICC = 0.85) and DR (ICC = 0.89) at 84% and 88%, respectively. IR and FA imaging offered the highest detection rates among the tested modalities, with 97% and 100%, respectively. Among the retro-mode, RA was highly efficient in depicting the lesions, with a 96% detection rate, and ICC = 0.97 (Figure 7, Table 2). Interoperator ICC was also high, with the lowest values found for DL.

## 4. Discussion

In this study, our findings underscore the utility of the novel retro-mode as an effective technique for identifying prevalent retinal conditions, including the presence of drusen, CSR, PED, or cRORA. Our research revealed the high sensitivity of this modality, particularly in the precise detection of retinal drusen. On the other hand, the RA mode demonstrated good sensitivity in detecting retinal atrophy. Although retro-mode has been on the market for some time, it remains unclear whether this method is superior to more established modalities [8]. One study investigated the use of retro-mode imaging to quantifying drusen in AMD. Compared to standard fundus photography, retro-mode imaging detected significantly more subretinal deposits (mean 81.6 vs. mean 33.5). The method also showed good agreement between graders and corresponded well with drusen observed through optical coherence tomography (OCT) imaging [18]. Another study emphasized the effectiveness of retro-mode imaging in quantifying geographic atrophy (GA) in patients with non-neovascular age-related macular degeneration (AMD). When compared to established imaging modalities like c-CFP, G-FAF, and B-FAF, retro-mode imaging showed good agreement in measuring the GA area. It demonstrated high inter-modality correlation and excellent agreement between graders, confirming its accuracy and reproducibility. Moreover, the use of infrared light in retro-mode imaging enhanced patient comfort. These findings suggested that retro-mode imaging could be a valuable tool for GA assessment in clinical settings [19]. 

In their study, Giansanti et al. employed retro-mode imaging in conjunction with FAF to identify outer retinal characteristics in patients with CSR. Their research demonstrated that retro-mode exhibited higher sensitivity compared to FAF in detecting specific CSCR features, although it displayed slightly lower specificity [20].

These findings suggest the potential for augmenting CSCR assessments by integrating retro-mode with other imaging modalities such as OCT and FAF [20]. Similarly, Cozzi et al. found that retro-mode imaging technology detected significantly more drusen-like deposits (DLDs) in the macula of healthy individuals compared to color fundus photography. The results showed that retro-mode imaging detected significantly more DLDs within the standardized early treatment diabetic retinopathy study (ETDRS) grid than color fundus photography (FP). At least one DLD was present in 96.71% of eyes on retro-mode, while only 63.74% of eyes showed DLDs with color FP. The density of DLDs increased with age, especially in the outer and inner rings of the macula. Retro-mode imaging shows promise as a non-invasive tool to investigate age-related changes in the macula [21]. In addition to its use in other conditions, retro-mode imaging was also employed in the evaluation of central serous chorioretinopathy (CSCR) using FAF, retro-mode, and enface imaging. RM imaging showed high specificity (91.7%) and negative predictive value (84.6%) in detecting retinal pigment epithelium (RPE) changes compared to AF. RM imaging could be a valuable adjunctive method in CSC evaluation [22]. 

In a study aimed at evaluating drusen detection in non-neovascular age-related macular degeneration (AMD), researchers compared retro-mode with color fundus photography. They employed various retro-mode and differential contrast (DC) techniques for this assessment. The results revealed a superior drusen detection rate with the employment of DR and DL when compared to color fundus photography. Interestingly, the application of DC did not lead to a significant enhancement in drusen detection, while DR and DL demonstrated a notably superior drusen detection capability compared to color fundus photography.

Consequently, the authors concluded that integrating retro-mode into clinical practice may prove invaluable for monitoring disease progression and evaluating the response to therapies in non-neovascular AMD cases. Additionally, this study highlighted a high level of agreement among graders when employing retro-mode, underscoring its reliability as an assessment tool [23]. It is important to note that this study was conducted on an older Nidek machine with a lower resolution than Mirante. 

Another study analyzed retro-mode imaging in detecting retinal changes secondary to exudative AMD. Retro-mode imaging showed excellent agreement in detecting cystoid macular edema associated with CNV, making it a useful and reproducible technique for identifying this feature. However, this was a relatively small study of 17 eyes [10]. 

Retro-mode proved a valuable technique in detecting choroidal nevi. The results showed that retro-mode imaging detected choroidal nevi with a characteristic “hypo-retro-reflective” pattern, even in cases not visible on other imaging modalities. It provided clear delineation of lesion margins with high sharpness and accuracy. The findings indicate that retro-mode scanning laser ophthalmoscopy is an innovative and reliable tool for the fast and non-invasive detection and follow-up of choroidal nevi. Another positive feature of retro-mode is the ability to present the images to the patient, for whom a pseudo-3D image may be easier to understand than a standard color fundus photo.

It is important to note that one of the biggest drawbacks of confocal retinal imaging is their relative inability to clearly depict choroidal nevi; hence, using the retro-mode can be beneficial [14]. 

Although it is essential to acknowledge that OCT is considered the gold standard for assessing DME, retro-mode imaging stands out as a valuable tool in the early detection of DME. Furthermore, retro-mode and OCT exhibit a notable level of agreement in the evaluation of diabetic macular edema (DME). Moreover, it provides the capability to visualize the location and extent of macular edema and aids in the identification of leaking microaneurysms, which manifest as localized elevations [24]. These findings are particularly significant because in late-phase fluorescein angiography (FA), these localized elevations correspond to microaneurysms with substantial dye diffusion [25]. Retro-mode imaging may be beneficial in examining neovascular vessels and fibrovascular membranes as well as identifying specific retinal changes [26]. Retro-mode has found application in the characterization of macular retinoschisis in highly myopic eyes. Retro-mode imaging showed a distinct fingerprint pattern in the area of retinoschisis, and the findings correlated well with conventional OCT. This suggests that retro-mode imaging has the potential to be a valuable tool for detecting and studying macular retinoschisis in clinical practice [27]. Another possible application of retro-mode is hydroxychlorchinine macular toxicity, with the typical feature being circular or ring-like regions around the fovea or in the near-central area that exhibit reduced reflectance and have visible deep choroidal vessels [28].

Among the tested imaging methods, we found high agreement between graders, with ICC values consistently close to or at 1.00 indicating excellent agreement. Only RA showed medium grading agreements between the graders. We can speculate that such low correlation comes from the novelty of this technique and lower experience compared with more established ones. 

There are three different options of retro-mode; in this study, we showed that RA mode is most useful for depicting atrophy, while changes that lead to a local increase in retinal thickness were best depicted using DL or DR modes. 

In our study, we found that the accuracy of FAF was approximately 85%, a result that aligns well with prior research indicating that FAF, on average, exhibits lower accuracy, e.g., in diagnosing Macular Telangiectasia (MacTel), when contrasted with multimodal imaging. These collective findings strongly suggest that the most straightforward approach to enhance the precision of retinal diagnoses is through the adoption of multimodal imaging techniques [29]. Another study aimed to analyze the contribution of retro-mode and fundus FAF in characterizing retinal dystrophies. The primary outcome was the identification of abnormal patterns on RMI, while the secondary outcome was the correlation of retro-mode findings with FAF-B and IR. Retro-mode revealed a distinct pseudo-3D pattern of lesions at the posterior pole, with material accumulation appearing as elevated areas with irregular and darker borders. However, the authors found no precise correlations between RMI, BL-FAF, and NIR-FAF. 

Nidek Mirante was also validated against the Heidelberg Engineering Spectralis in a study that investigated the reliability and comparability of retinal measurements using these two different imaging platforms (Heidelberg Spectralis and Nidek Mirante) in eyes with macular pathology and healthy volunteers. The results showed no significant differences in retinal measurements between the devices. Common ultrastructural biomarkers of various macular pathologies were identified with high sensitivity and specificity, indicating comparable confidence in retinal imaging between the two devices. These findings support the interchangeability of the two platforms for clinical use [13]. Another intriguing facet of our study lies in the potential to diminish the necessity for performing FA or OCT procedures. Consequently, retro-mode imaging may present itself as a cost-effective alternative to these more advanced imaging methods while retaining its capacity to deliver clinically valuable information. Limitations: Several limitations were identified in this study. Firstly, the sample size may not be representative of the entire population, potentially limiting generalizability. Secondly, the study focused on specific ocular conditions, and results may not apply to other pathologies. Thirdly we did not differentiate between different types of drusen. Finally, variations in operator technique and patient factors could introduce bias or variability in the imaging results [30].

## 5. Conclusions

Retro-mode imaging on Nidek Miarante stands out for its superior visualization of specific retinal structures, such as retinal drusen, when compared to traditional color fundus photography and FAF imaging techniques. The non-invasive and rapid nature of Retro-mode imaging underscores its potential as a valuable tool for detecting conditions like PED, drusen, and AMD. Furthermore, it proves to be an effective means for tracking disease progression and evaluating responses to therapeutic interventions.

Notably, both DR and DL demonstrate excellence in capturing detailed images of drusen, while RA excels in imaging atrophy. Nevertheless, it is imperative to emphasize the need for more extensive studies to conclusively establish its utility. In light of the findings from this study, it becomes evident that, for a majority of retinal cases, a multimodal imaging approach emerges as the most beneficial approach.

## Figures and Tables

**Table 1:** Ocular parameters of the study population (*n* = 115).

Variable	Mean	Std. Dev.
BCVA	0.53	0.34
Cylinder Refraction	−0.84	0.51
Refraction sphere	23.53	42.54
Axis	88.30	54.48
IOP	15.41	1.94

**Table 2:** Intraclass correlation coefficient (ICC) values for various imaging modalities and their comparison between two graders (A vs. B) as well as in detecting different features (drusen, SRF, PED, and cRORA).

	Drusen		CSR		PED		cRORA	
	ICC vs. OCT	ICC A vs. B	ICC vs. OCT	ICC A vs. B	ICC vs. OCT	ICC A vs. B	ICC vs. OCT	ICC A vs. B
DL	0.96	0.89	1	1	1	0.98	0.85	0.81
DR	0.94	0.91	1	1	1	1	0.89	0.9
RA	0.39	0.83	0.5	0.75	0.5	0.8	0.97	0.93
FA	0.73	1	1	1	1	1	1	1
Color	0.43	0.89	0.69	0.87	0.91	0.91	0.95	0.88
OCT	1	1	1	1	1	1	1	1
IR	0.73	0.97	0.78	1	0.96	0.84	0.99	0.97
FAF-G	0.49	0.91	0.79	1	0.97	0.94	0.89	0.88
FAF-B	0.47	0.91	0.78	1	0.79	0.89	0.91	0.84

Retinal pigment epithelium and ocular retina atrophy (cRORA). Pigment epithelial detachment (PED), central serous chorioretinopathy (CSR). Fundus autofluorescence green (FAF-G) grading; fundus autofluorescence–blue-scan (FAF-B); three-dimensional optical coherence tomography (3D-OCT); color imaging; Retromode aperture deviated to the left (DL); Retromode aperture deviated to the right (RA); Retromode ring aperture (DR); fluorescein angiography (FA); infrared imaging (IR); optical coherence tomography (OCT).

## Data Availability

Data are available upon reasonable request from the corresponding author.
